# Hydroxychloroquine inhibits CD154 expression in CD4^+^ T lymphocytes of systemic lupus erythematosus through NFAT, but not STAT5, signaling

**DOI:** 10.1186/s13075-017-1393-y

**Published:** 2017-08-09

**Authors:** Shu-Fen Wu, Chia-Bin Chang, Jui-Mei Hsu, Ming-Chi Lu, Ning-Sheng Lai, Chin Li, Chien-Hsueh Tung

**Affiliations:** 1 Department of Life Science, Institute of Molecular Biology, National Chung-Cheng University, No.168, University Rd, Min-Hsiung, Chia-Yi, 62247 Taiwan; 2 Division of Allergy, Immunology and Rheumatology, Dalin Tzu Chi Hospital, Buddhist Tzu Chi Medical Foundation, Chia-Yi, Taiwan; 3 College of Medicine, Tzu Chi University, Hualien, Taiwan; 4 Department of Medical Research, Dalin Tzu Chi Hospital, Buddhist Tzu Chi Medical Foundation, Dalin, Chia-Yi, Taiwan

**Keywords:** CD154, Hydroxychloroquine, Systemic lupus erythematosus, NFAT, STAT5

## Abstract

**Background:**

Overexpression of membranous CD154 in T lymphocytes has been found previously in systemic lupus erythematosus (SLE). Because hydroxychloroquine (HCQ) has been used frequently in the treatment of lupus, we sought to identify the effects of HCQ on CD154 and a possibly regulatory mechanism.

**Methods:**

CD4^+^ T cells were isolated from the blood of lupus patients. After stimulation with ionomycin or IL-15 and various concentrations of HCQ, expression of membranous CD154 and NFAT and STAT5 signaling were assessed.

**Results:**

HCQ treatment had significant dose-dependent suppressive effects on membranous CD154 expression in ionomycin-activated T cells from lupus patients. Furthermore, HCQ inhibited intracellular sustained calcium storage release, and attenuated the nuclear translocation of NFATc2 and the expression of NFATc1. However, CD154 expressed through IL-15-mediated STAT5 signaling was not inhibited by HCQ treatment.

**Conclusions:**

HCQ inhibited NFAT signaling in activated T cells and blocked the expression of membranous CD154, but not STAT5 signaling. These findings provide a mechanistic insight into SLE in HCQ treatment.

## Background

Systemic lupus erythematosus (SLE) is a systemic autoimmune disease that has a complex immunological pathogenesis, mainly associated with autoantibody synthesis. Antibody isotype switch and affinity maturation is induced through T-cell-dependent B-cell activation [1], involving interactions between cell surface CD154 on CD4^+^ T-helper (Th) cells and CD40 on B cells [2, 3]. Blockade of the CD154/CD40 interaction inhibited T-cell-dependent B-cell proliferation, differentiation, and antibody formation in in-vitro studies and in lupus mouse models [4–6]. Thus, rigorous regulation of Th cell CD154 expression is necessary to maintain the antigen specificity and antibody synthesis of the immune response [7–10].

Overexpression of cell surface CD154 on CD4^+^ T cells was found in patients with SLE [5, 11–15]. The surface CD154 levels before and after stimulation both increased in active lupus patients when compared with healthy controls or remission lupus patients [15]. The active CD154 expression was associated with SLE disease severity, lupus nephritis, and antibody synthesis [14–16]. Furthermore, CD154 gene promoter activity is activation dependent, regulated by the calcineurin-dependent transcription factors of nuclear factor of activated T cells (NFAT), including NFATc2 (NFAT-1) in the early phase and NFATc1 (NFAT-2) in the late phase [3, 7, 10, 14]. Thus, the production of membranous CD154 is mainly mediated through Ca^2+^-NFAT signaling pathways [17, 18]. In SLE, increased CD154 expression was associated with enhanced intracellular Ca^2+^ signaling response, NFAT overexpression, NFAT nuclear translocation, and NFAT-mediated CD154 gene transcription [10, 14, 19, 20].

In addition, prolonged CD154 expression is also associated with the interleukin-15 (IL-15) signaling pathway [21]. After IL-15 binding with its receptor, this process activates Jak1 and Jak3 and subsequently phoshorylates STAT5. The phosphorylated STAT5 translocates to the nucleus, engages the CD154 gene promoter, and induces CD154 expression. Overproduction of IL-15 by T cells was observed in SLE patients [22, 23]. IL-15 signaling is therefore another pathway which contributes to CD154 overexpression in SLE. Increased Th cell surface expression of CD154 is reflected in an enhanced capacity to mediate B-cell activation, antigen expression, and autoantibody production [24, 25]. The involvement of the CD40–CD154 interaction pathway in the pathogenesis of SLE has been reported in several studies using human and mouse models [26–28].

Hydroxychloroquine (HCQ) was first used for its anti-malaria activity, but has subsequently been used as an anti-rheumatic drug, and is frequently prescribed for SLE patients [29]. HCQ can attenuate SLE disease severity, inflammatory parameters, and immunoglobulin levels in treated patients [30–32]. HCQ mainly accumulates in acidic vesicular lysosomes. In antigen-presenting cells, HCQ interfered with the function of the proteosome by increasing the pH of intracellular vacuoles, and also altered the bioactivity of many intracellular proteins [29, 33]. Thus, HCQ blocked antigen presentation and immune synapse between T cells and antigen-presenting cells, and thereafter blocked downstream immune responses [34]. Moreover, in T cells, HCQ has been shown to attenuate T-cell proliferative responses to mitogens, while reducing the release of proinflammatory cytokines through inhibiting Toll-like receptor pathways [33, 35]. Furthermore, HCQ also blocked the T-cell activation pathway by interfering with intracellular calcium signaling [36]. Thus, the diverse immune modulatory effects of HCQ make it widely used in treatment of autoimmune diseases.

CD154 overexpression is associated with enhanced NFAT and STAT5 signaling. It is critical for autoantibody production in SLE patients. Because the overinteraction of CD40–CD154 is detrimental in autoimmune diseases, it had been of great interest in developing targeting therapy [27, 37]. HCQ is the most common medication used in SLE. However, to date there was no previous report about the effect of HCQ on CD154 expression. In this study, we investigated whether HCQ modulated the important membrane protein of CD154 expression in SLE T lymphocytes and its possible underlying mechanisms of action.

## Methods

### Patients

Eight healthy control individuals and 27 patients with systemic lupus erythematosus (SLE) who fulfilled at least four of the revised criteria of the American College of Rheumatology were enrolled in this study [38]. The clinical characteristics of the study cohorts are presented in Table 1. SLE disease activity was evaluated by the SLE Disease Activity Index (SLEDAI). The patients with SLEDAI score > 9 were defined as having high disease activity [39]. A total of 20 ml of blood was sampled from each patient for various experiments.

**Table 1:** Demographic data of SLE patients (*n* = 27)

Clinical characteristic	Demographic data
Age (years)	33.9 ± 13
Sex, female:male	26:1
Duration (years)	7.7 ± 7.2
SLEDAI score	11.8 ± 9.2
Major organ involvement	
Nephritis	48% (13/27)
Cytopenia	22% (6/27)
Central nervous system	22% (6/27)
Immunosuppressants	
Corticosteroid	100%
Hydroxychloroquine	7%
Azathioprine	33%
Mycophenolate mofetil	40%
Cyclophosphamide	11%
Immunological level	
dsDNA Ab	108.1 ± 148.7
C3	74.4 ± 34.4
C4	14.7 ± 10.6
ACA-IgG	10.1 ± 13.1
ESR	49.2 ± 51.5

Data presented as mean ± standard deviation or percentage (number/total)

*SLE* systemic lupus erythematosus, *SLEDAI* SLE Disease Activity Index, *ESR* erythrocyte sedimentation rate, *dsDNA Ab* anti-double-strand DNA antibody, *ACA-IgG* anti-cardiolipin IgG

### Cell separation

Human peripheral blood mononuclear cells were isolated from sampled heparinized fresh venous blood of SLE donors or healthy controls using Ficoll-paque density gradient centrifugation. In short, 20 ml of fresh blood was collected and spun at 2000 rpm for 10 min at room temperature. Blood plasma (upper fraction) was discarded, while blood cells in the lower fraction were resuspended in an equal volume of phosphate-buffered saline, followed by overlay onto a half volume of Ficoll-paque Plus (17-1440-03; GE Healthcare, Amersham, UK). Cells were centrifuged at 2000 rpm for 20 min at room temperature, resulting in the generation of the peripheral blood mononuclear cell fraction between serum and Ficoll-paque fractions for collection. Subsequently, purified CD4^+^ T cells were negatively isolated from peripheral blood mononuclear cells by CD4^+^ T-cell isolation kits (19052; STEMCELL Technologies, Vancouver, Canada) according to the manufacturer’s protocol. In brief, peripheral blood mononuclear cells were washed twice, counted, and resuspended in 2–5 mM cold EDTA isolation buffer at a cell density of 1 × 10^7^ cells/200 μl. Cells were mixed with 10 μl of biotin-Ab cocktail (Abs for cells other than CD4^+^ T cells) and incubated for 10 min at 4 °C, followed by the addition 20 μl of anti-biotin microbeads. The cell–Ab–microbead reaction mixture was then incubated for 10 min at 4 °C, centrifuged, and cells resuspended in 2 ml of 2.5 mM cold EDTA buffer. For CD4^+^ T-cell purification, the cell mixture was added through a LS (MACS) column to exclude the cells other than CD4^+^ T cells.

### Medium, buffer, and reagents

Purified T cells were cultured in RPMI-1640-based medium (Gibco/BRL, Carlsbad, CA, USA) supplemented with 1% l-glutamine, 100 U/ml penicillin, 100 U/ml streptomycin, 10% fetal bovine serum (Hyclone, Logan, UT, USA), 10 mM HEPES, and 50 μM β-mercaptoethanol. Jurkat T cells (clone E6-1) were maintained in complete RPMI medium (10% fetal bovine serum, 100 U/ml penicillin, 100 U/ml streptomycin, and 2 mM glutamine). The isolation buffer was phosphate-buffered saline supplemented with 2 mM EDTA and 2% FBS. The hypotonic RBC lysis buffer consisting of 0.15 M NH_4_Cl, 10 mM KHCO_3_, and 0.1 mM EDTA was used to adjust the pH to 7.2, followed by sterilization through 0.22-μm filters and storage at room temperature. Hydroxychloroquine was purchased from Sanofi Pharmaceuticals (Paris, France) and dissolved in sterile water at a concentration of 600 ng/ml (the standard concentration) and stored at −80 °C [30, 40]. In the ionomycin study, ionomycin (I3909; Sigma, St. Louis, MO, USA) was used at 1 or 5 μg/ml while phorbol 12-myrisate 13-acetate (PMA) (P8139; Sigma) was used at 50 ng/ml for T-cell stimulation. In the IL-15 study, cells were stimulated with 1.5 μg/ml polyhydroxyalkanoates (PHA) (L1668; Sigma), 75 ng/ml IL-15 alone (247-ILB; R&D Systems, Minneapolis, MN, USA), or a combination.

### In-vitro HCQ treatment of CD4^+^ T cells and Jurkat cells

The purified CD4^+^ T cells were isolated by negative selection because positive selection may induce T-cell activation before the experiment. The isolated CD4^+^ T cells were diluted to a concentration of 1 × 10^6^/ml. We use only SLE samples because the poststimulated CD154 overexpression is critical and more obvious in SLE patients than in healthy controls [14, 17]. Jurkat cells were also diluted to a concentration of 1 × 10^6^/ml for further experiments. The experimental cells, 2 × 10^5^ cells/200 μl, were grown in 96-well tissue culture plates, and then incubated for 24 hours in the presence of various concentrations of HCQ (1 × = 600 ng/ml). Ionomycin is known to cause sustained Ca^2+^ storage release from the endoplasmic reticulum and to activate intracellular Ca^2+^ signaling, which is important for CD154 transcription [41]. In the ionomycin study, cells were activated by different concentrations and durations of ionomycin, depending upon the specific experiment. In the IL-15 study, IL-15, PHA, or a combination were used to stimulate T cells. We used stimulation duration of 6 or 20 hours in HCQ inhibition experiments because significant increase of CD154 expression was seen at this stimulation duration in previous studies and our results [14, 21, 42]. After stimulation, cells were stained for CD4, CD154, or NFATc1, according to the manufacturer’s guidelines, and analyzed by flow cytometry.

### Flow cytometric analysis

Flow cytometric analysis was performed using a FACS Calibur flow cytometer and CellQuest software (BD Biosciences). The cells were washed and subsequently incubated with the indicated anti-cell surface antibody diluted in 200 μl of blocking buffer at 4 °C for 30 min, spun, and then washed twice with cold phosphate-buffered saline. When staining for intracellular markers, cells were washed, fixed, and permeabilized with commercial fixed/permabilization buffer (00-5123; eBioscience) at 4 °C for 30 min, and then incubated with the indicated antibody at 4 °C for 30 min, spun, and washed twice with cold PBS. Cell staining for flow analysis was performed with the following specific antibodies: anti-human CD4–FITC (catalog no. 555346) and anti-human CD154-PE (catalog no. 555700), purchased from BD Pharmingen. Anti-human NFATc1-PE (lot no. D0612) was purchased from Santa Cruz Biotechnology (Santa Cruz, CA, USA).

### Cell viability assay

Cell viability after culturing in the presence of HCQ was assessed by MTT assay, following the manufacturer’s instructions (Sigma Chemical Co.). In brief, cells were grown for 6 hours in 96-well tissue culture plates (2 × 10^5^ cells/200 μl) and incubated with HCQ in various concentrations. MTT (dimethylthiazolyl-2-5-diphenyltetrazolium bromide) dye solution (Omega, St. Louis, MO, USA) was added 24 hours later and incubated at 37 °C for 2 hours; MTT was reduced by live cells into a colored product. Absorbance at 492 nm wavelength was recorded using Thermo Scientific Multiskan™ GO (Thermo Scientific, Waltham, MA, USA). Each treatment was repeated in quadruplicate. Cell viabilities were defined relative to control cells which were not treated with HCQ, with results used as evaluation of the cytotoxicity of HCQ. The half-maximal cytotoxic concentration (CC50) for each compound was calculated from the dose–response curves with the aid of GraphPad Prism software 5.01 (GraphPad Software, San Diego, CA, USA).

### Analysis of intracellular free calcium concentration

Cultured Jurkat T cells, 1 × 10^5^ cells /100 μl, were incubated with different concentrations of HCQ (1 × = 600 ng/ml) or PBS for 24 hours. Fluo-4 Direct™ Calcium Assay Kits (catalog no. F10472; Invitrogen, Carlsbad, CA, USA) were used for measuring the intracellular free calcium concentration. In brief, cells were harvested, precultured with the indicated buffer for 1 hour, and then loaded with the calcium-sensitive indicator dye Fluo-4 Direct™ reagent at 37 °C for 30–60 min following the manufacturer’s protocol. Cells were kept at 4 °C and protected from light prior to analysis. Fluorophore-loaded cells were then stimulated with 1 μg/ml ionomycin and immediately monitored at different time points after stimulation. Intracellular calcium measurements were fluorescently monitored at 494 nm excitation and 516 nm emission by means of a bulk spectrofluorometer (Multiskan™ GO; Thermo Scientific, Waltham, MA, USA).

### Nuclear and cytoplasmic protein extraction and western blot analysis

Cell extracts were harvested for western blot analysis after treatments depending upon the specific experiments. In the ionomycin study, the Jurkat cells, 1 × 10^5^ cells/100 μl, were stimulated with 1 μg/ml ionomycin for 2 hours after being pretreated with HCQ for 24 hours. In the IL-15 study, after pretreatment with HCQ for 24 hours, the Jurkat cells were then stimulated with 20 ng/ml IL-15 for 20 min. The cells after stimulation were treated initially on ice with a 200 μl lysis buffer (10 mM HEPES (pH 7.9), 10 mM KCl, 0.1 mM EDTA, 0.1 mM EGTA supplemented with freshly added 1 mM DTT, 0.5 mM PMSF, 2 mM aprotinin, 1 mM leupeptin, 10 mM NaF, and 2 mM Na_3_VO_4_) for 15 min. In the IL-15 study, cell extracts were harvested after centrifugation. In the ionomycin study, at the end of the incubation, Nonidet P-40 was added to the reaction mixture at a concentration of 0.6%. The reaction mixture was vortexed for 10 sec and then subjected to centrifugation at 13,000 rpm for 15 sec. The supernatant was saved as cytoplasmic extract. The pellet was resuspended in 25 μl of buffer (20 mM HEPES (pH 7.9), 0.4 M NaCl, 1 mM EDTA, 1 mM EGTA, 10 mM NaF, 1 mM Na_3_VO4, 1 mM PMSF, 2 mM aprotinin, and 1 mM leupeptin) and then shaken for 15 min at 4 °C. After centrifugation for 5 min at 13,000 rpm, the supernatant was stored as nuclear extract (CelLytic NuCLEAR Extraction Kit; Sigma). We followed the manufacturer’s instructions (ECL; Amersham) for the western blot analysis. Anti-human NFATc2 (2172755; Millipore Biotechnology) and anti-human phosphorylated STAT5 (9351; Cell Signaling) were used. The film was scanned, and the density of each band was calculated with QuantityOne software (Bio-Rad).

### Immunocytochemistry

Purified T cells were treated without or with HCQ of indicated concentration for 24 hours and then stimulated with ionomycin (1 μg/ml) for 20 min. Cells (1 × 10^6^) were transferred onto the glass and fixed by incubation with ice-cold 100% methanol for 10–15 min at room temperature. After washing with PBS (0.25% Triton X-100), cells were blocked with PBST (PBS with 0.1% Tween 20 and 3% BSA) for 30 min at 37 °C. Cells were incubated with anti-human NFATc2 antibody (2172755; Millipore Biotechnology) for 12 hours at 4 °C and then stained with rhodamine-conjugated anti-mouse IgG Ab. Stained cells were examined using a FV1000 confocal microscope (Olympus), and images were processed using Olympus FLUOVIEW software.

### Statistical analysis

The measured items and domain scores for the study groups were presented as mean ± standard deviation. All statistical tests were performed using SPSS-19 software (IBM, Amonk, NY, USA). The flow cytometric data of in-vitro cell culture systems did not fit a Gaussian distribution. Therefore, nonparametric Kruskal–Wallis tests were used to analyze these data to determine whether there were significant differences in the medians of the groups analyzed. If 95% significance was achieved, a paired *t* test was then used to compare the assay results of one group with another. In all cases, *p* < 0.05 (two sided) was considered statistically significant. Data from in-vitro cell culture systems were expressed as mean ± SEM of at least three independent experiments, performed in triplicate.

## Results

### Poststimulated membrane CD154 was overexpressed in SLE patients and associated with SLE disease severity

**Fig. 1:**
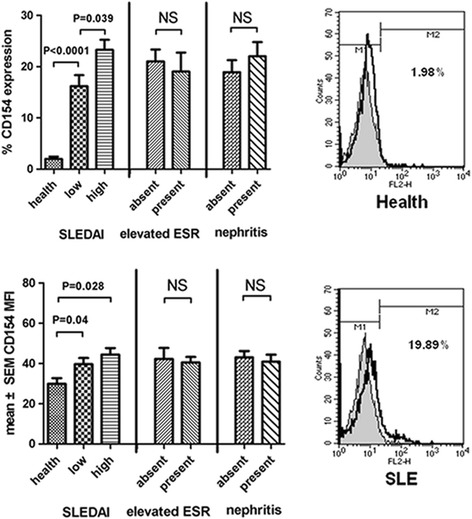
Poststimulated membrane CD154 was overexpressed in SLE patients and associated with SLE disease severity. Level of CD154 expression on purified CD4^+^ T cells examined by flow cytometry after 6 hours of 1 μg/ml ionomycin activation in relation to the healthy controls (*n* = 8) or lupus patients (*n* = 20), low or high SLE disease activity, presence or absence of nephritis, or an elevated ESR. Definition of high disease activity was SLEDAI score > 9. Definition of nephritis was the presence of proteinuria (daily urine protein > 0.5 g/day) closest to the time of analysis. *MFI* mean fluorescence intensity, *SLEDAI* SLE Disease Activity Index, *ESR* erythrocyte sedimentation rate, *NS* not significant, *SLE* systemic lupus erythematosus

Previous studies have shown that post-stimulated CD154 expression is clinically associated with ESR and lupus nephritis, but not medications or levels of autoantibodies in SLE patients [14, 15]. The CD154 overexpression in SLE was associated with disease severity [15]. We investigated the clinical association with poststimulated CD154 expression level. The CD154 expression on purified CD4^+^ T cells from healthy control and SLE patients was analyzed by flow cytometry after ionomycin stimulation for 6 hours. The poststimulated CD154 expression level (percentage and MFI) was significantly higher in SLE patients when compared with healthy controls (Fig. 1). Besides, the percentage of the CD154-expressing cells was significantly higher in lupus patients with high disease activity (SLEDAI score > 9) than in those with lower disease activity (Fig. 1) [39]. Therefore we used purified T cells from SLE patients for the next experiments. Furthermore, the percentage of CD154-expressing cells was higher in patients with lupus nephritis than in those without nephritis, but not significantly (Fig. 1). In addition, the CD154 level did not correlate with age, clinical manifestations, medications, ESR, or autoantibodies.

### HCQ treatment has dose-dependent suppressive effects on poststimulated membranous CD154 expression

**Fig. 2:**
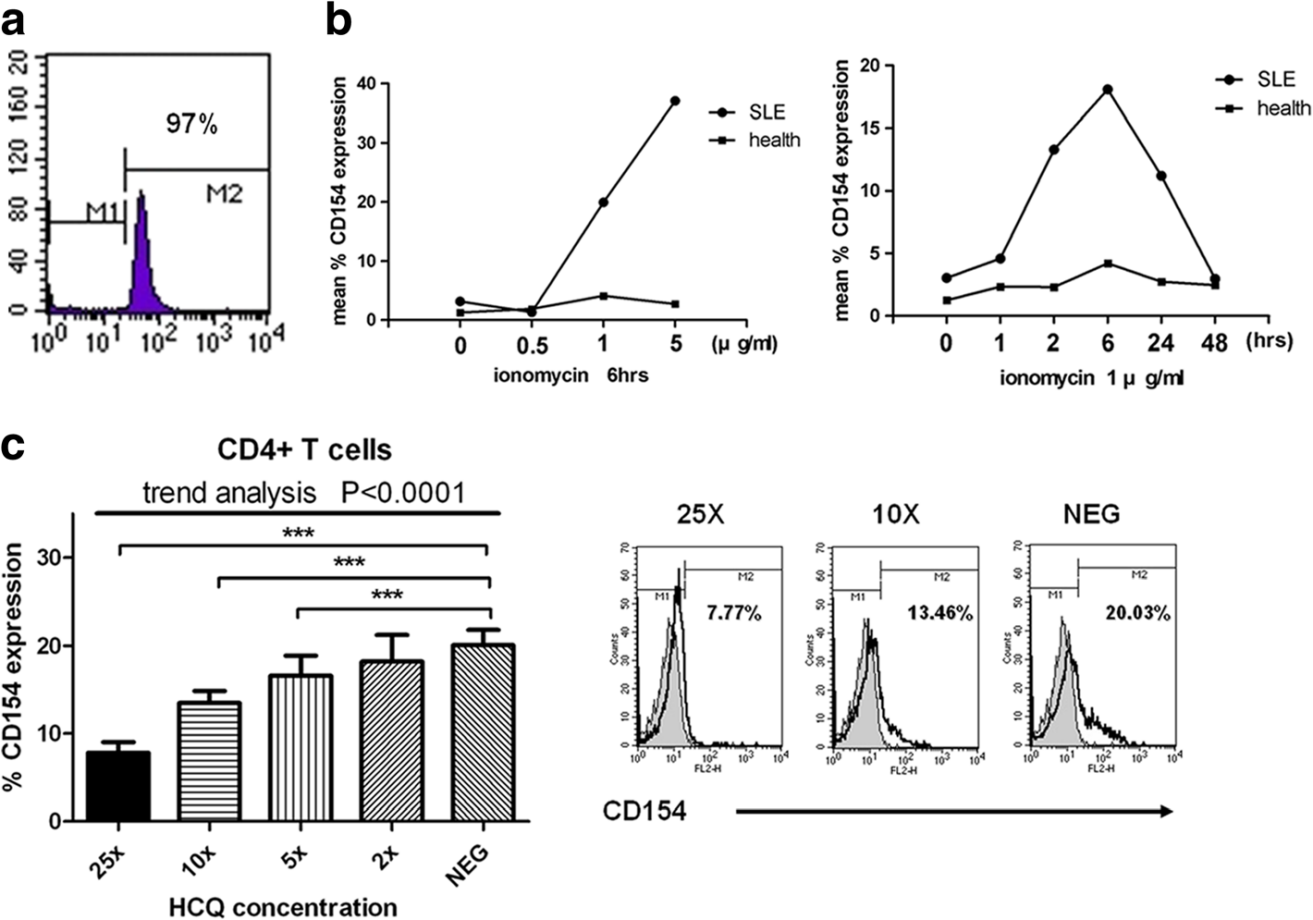
Purified CD4^+^ T cells from SLE patients upregulate CD154 after stimulation, while HCQ downregulates CD154. **a** Purity of the isolated CD4^+^ T cells was 97%. **b** Purified CD4^+^ T cells from SLE patients (*n* = 4) and healthy controls (*n* = 4) were treated with various concentrations of ionomycin for 6 hours (*left panel*) or 1 μg/ml ionomycin for different time periods (*right panel*). CD154 expression was measured by flow cytometry. **c** CD4^+^ T cells were purified from SLE patients and cultured in different concentrations of HCQ (1 × = 600 ng/ml), as indicated, and activated with ionomycin (1 μg/ml) for 6 hours (*left panel*). Flow cytometry was used to measure CD154 expression (*n* = 11). ***p < 0.005. Histogram plots of flow cytometry analysis with various concentrations of HCQ are indicated (*right panel*). *HCQ* hydroxychloroquine, *SLE* systemic lupus erythematosus, *NEG* no HCQ treatment

The previous report revealed that membranous CD154 and soluble CD154 expression in T cells was regulated by different intracellular signal pathways [39]. The membranous CD154 expression mainly depended on the intracellular Ca^2+^ signaling pathway, which can be stimulated by ionomycin [10, 41]. Because PMA or anti-CD3/CD28 stimulation caused membranous CD154 shedding from cells and attenuated the membranous CD154 expression, we therefore used ionomycin stimulation alone to focus on the Ca^2+^ signaling pathway. Purified CD4^+^ T cells were used for the next experiments to avoid secondary effects of cells other than T cells. The purity was 92–97% (Fig. 2a). To verify the role of ionomycin on membranous CD154 expression, purified CD4^+^ T cells from SLE patients were treated with various concentrations of ionomycin. Our results showed that higher concentrations of ionomycin induced higher levels of membranous CD154 expression (Fig. 2b, left panel). Second, we treated the purified T cells with differing durations of ionomycin exposure, showing that longer ionomycin treatment further upregulated membranous CD154 (Fig. 2b, right panel). The results revealed that the concentration and duration of stimulation conditions was important in CD154 expression. Downregulation of CD154 was noted if the stimulation duration was longer than 24 hours. After 48 hours of stimulation, CD154 expression regained levels similar to those without stimulation. In addition, we performed the same experiments with purified T cells from healthy controls (Fig. 2b). Despite stimulation with various durations or concentrations of ionomycin, T cells from lupus patients expressed higher CD154 than healthy control cells.

To date, no previous study has reported a direct correlation between the membranous CD154 expression and the clinical use of HCQ. In order to assess whether HCQ had a dose-dependent inhibitory effect on CD154 expression in SLE patients, various doses of HCQ were used to treat purified CD4^+^ T cells from lupus patients in vitro for 24 hours, and then stimulated by 1 μg/ml ionomycin for 6 hours. The expression of CD154 on CD4^+^ T cells was attenuated gradually when the HCQ concentration was increased. The results showed that higher doses of HCQ had significantly higher inhibition effects on poststimulated membranous CD154 expression, especially when the concentration of HCQ was higher than 5× standard concentration (=3000 ng/ml) (Fig. 2c). Summarily, our results indicate that HCQ has a primary dose-dependent suppressive effect on membranous CD154 expression on CD4^+^ T cells from SLE patients.

### HCQ inhibitory capacity is specific and has limited cytotoxic effects

**Fig. 3:**
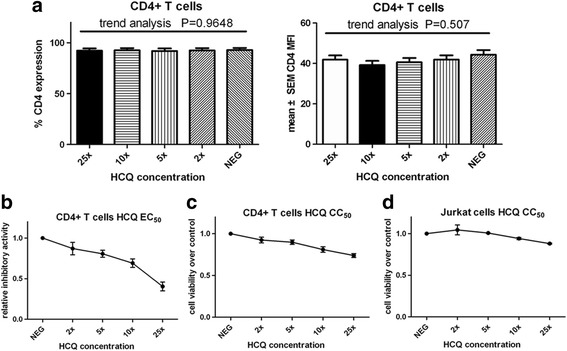
HCQ inhibitory capacity is specific and has limited cytotoxic effects. **a** Purified CD4^+^ T cells were cultured in different concentrations of HCQ (1 × = 600 ng/ml), as indicated, and activated with ionomycin (1 μg/ml) for 6 hours. Flow cytometry was used to measure CD4 expression (*n* = 9). **b** Effective concentrations of HCQ on CD154 expression were determined by the calculation of the half-maximal effective concentration (*EC50*) from the flow cytometric results. Inhibitory ability of HCQ relative to vehicle control was determined by plotting to determine the EC50. **c**, **d** CD4^+^ T cells and Jurkat cells were pretreated with various concentrations of HCQ (1 × = 600 ng/ml) for 24 hours and then loaded with a MTT reagent. After incubation for at least 2 hours, the absorbance at 492 nm was recorded by spectrofluorometry to obtain the half-maximal cytotoxic concentration (*CC50*) values. These results were shown as mean ± SEM and represent one assessment; more than six independent experiments had similar results. *HCQ* hydroxychloroquine, *NEG* no HCQ treatment

In the data already presented, we confirmed that HCQ treatment significantly decreased membranous CD154 expression. To investigate the specificity of HCQ effect on CD154 expression, we also evaluate the effect of HCQ on CD4, another membranous molecule of T cells. CD4 was constitutively expressed on the surface of CD4^+^ T cells. We used various concentrations of HCQ to treated purified CD4^+^ T cells for 24 hours and measured the CD4 expression level after stimulation with 1 μg/ml ionomycin for 6 hours. We found that HCQ did not inhibit the CD4 expression (percentage and MFI) (Fig. 3a). The inhibitory capacity of HCQ was specific to CD154.

The effective concentrations of HCQ on CD154 expression were defined by the calculation of the half-maximal effective concentration (EC50) from the previous results (Fig. 3b). The EC50 values were determined by plotting the logarithm of HCQ concentration versus relative inhibitory activity. Results showed that HCQ inhibited CD154 expression in CD4^+^ T cells with 20% reduction at 5× standard concentration (=3000 ng/ml) and more than 50% reduction at 25× standard concentration treatment (Fig. 3b).

However, there was a concern that the results may be due to nonspecific cellular toxicity of HCQ at high doses. Therefore, to determine the effect of the HCQ on cell viability and toxicity, purified CD4^+^ T cells or Jurkat cells were cultured with increasing concentrations of HCQ for 24 hours and MTT assays were performed to obtain the half-maximal cytotoxic concentration values (CC50) (Fig. 3c, d). We demonstrated that HCQ slightly inhibited T-cell and Jurkat cell viability with less than 10% reduction at 5× standard concentration (=3000 ng/ml) and less than 30% reduction over vehicle control at 25× standard concentration, indicating low toxicity of HCQ in those cells. Abrogating expression of CD154 by HCQ was therefore not due to nonspecific cellular toxicity of HCQ.

### HCQ inhibits CD154 expression through inhibition of sustained Ca^2+^ storage release from the endoplasmic reticulum

**Fig. 4:**
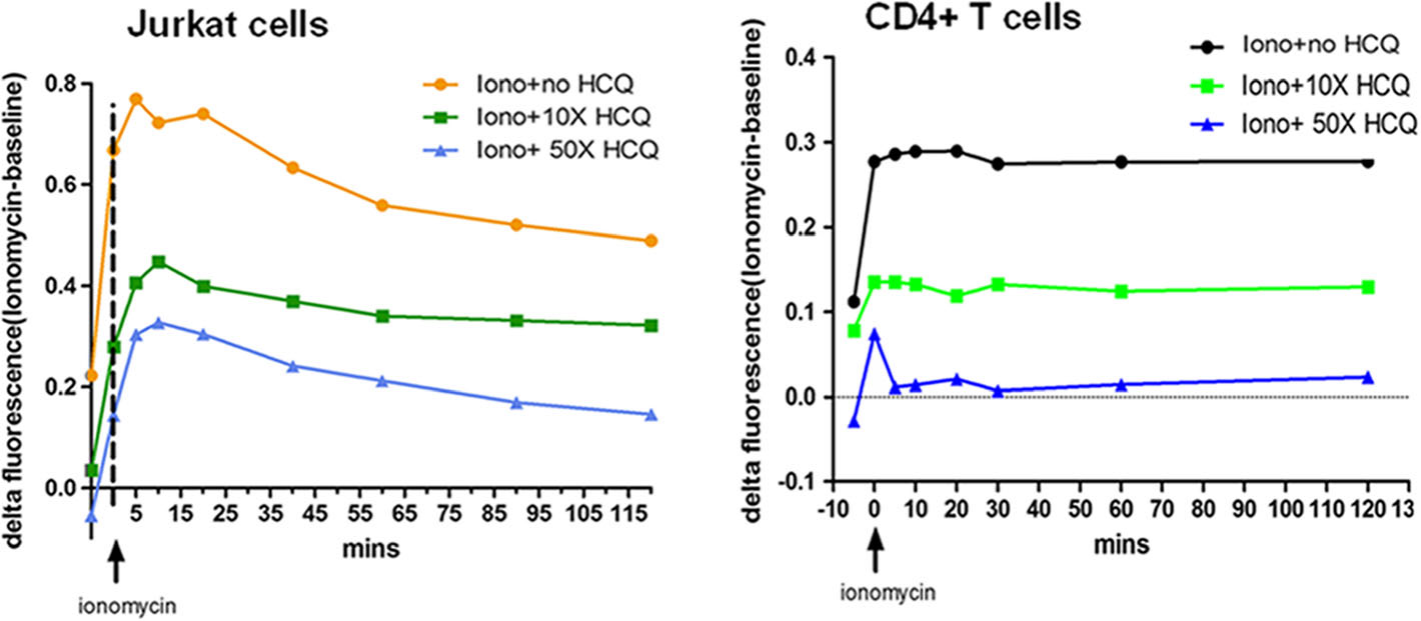
HCQ treatment inhibits ionomycin-dependent intracellular calcium mobilization. Jurkat cells (*left*) and purified CD4^+^ T cells (*right*) were pretreated with various concentrations of HCQ (1 × = 600 ng/ml) for 24 hours, loaded with a calcium-sensitive dye, and then activated by 1 μg/ml ionomycin (as indicated by the *arrow*). Before and after stimulation, changes of intracellular free Ca^2+^ concentration (*delta fluorescence*) versus time were monitored by spectrofluorometry at different time points. These results represent one assessment; more than six independent experiments had similar results. *HCQ* hydroxychloroquine

In the data already presented, we confirmed that HCQ treatment significantly decreased membranous CD154 expression after ionomycin stimulation, the inducer of intracellular Ca^2+^ response. The previous study demonstrated that HCQ inhibited the free Ca^2+^ exflux in Jurkat cell lines and T cells [36]. Therefore we performed in-vitro studies to validate whether HCQ could inhibit sustained Ca^2+^ storage release after ionomycin stimulation. In our study, various HCQ concentrations were used to treat Jurkat cells and purified T cells for 24 hours, and Ca^2+^ storage release was measured at different time points after ionomycin stimulation (1 μg/ml). Those results showed significant inhibition of Ca^2+^ release when HCQ of 10× standard concentration was used (=6000 ng/ml) (Fig. 4). Higher HCQ concentrations were associated with significantly lower Ca^2+^ release in a dose-dependent manner, as compared with vehicle-treated cells (Fig. 4). In addition, the inhibition effect of HCQ on calcium response lasts for 2 hours. Therefore, HCQ inhibits sustained Ca^2+^ storage release from the endoplasmic reticulum and subsequent Ca^2+^ signaling.

### In-vitro HCQ treatment decreases NFATc2 nuclear translocation and NFATc1 expression in CD4^+^ T cells

**Fig. 5:**
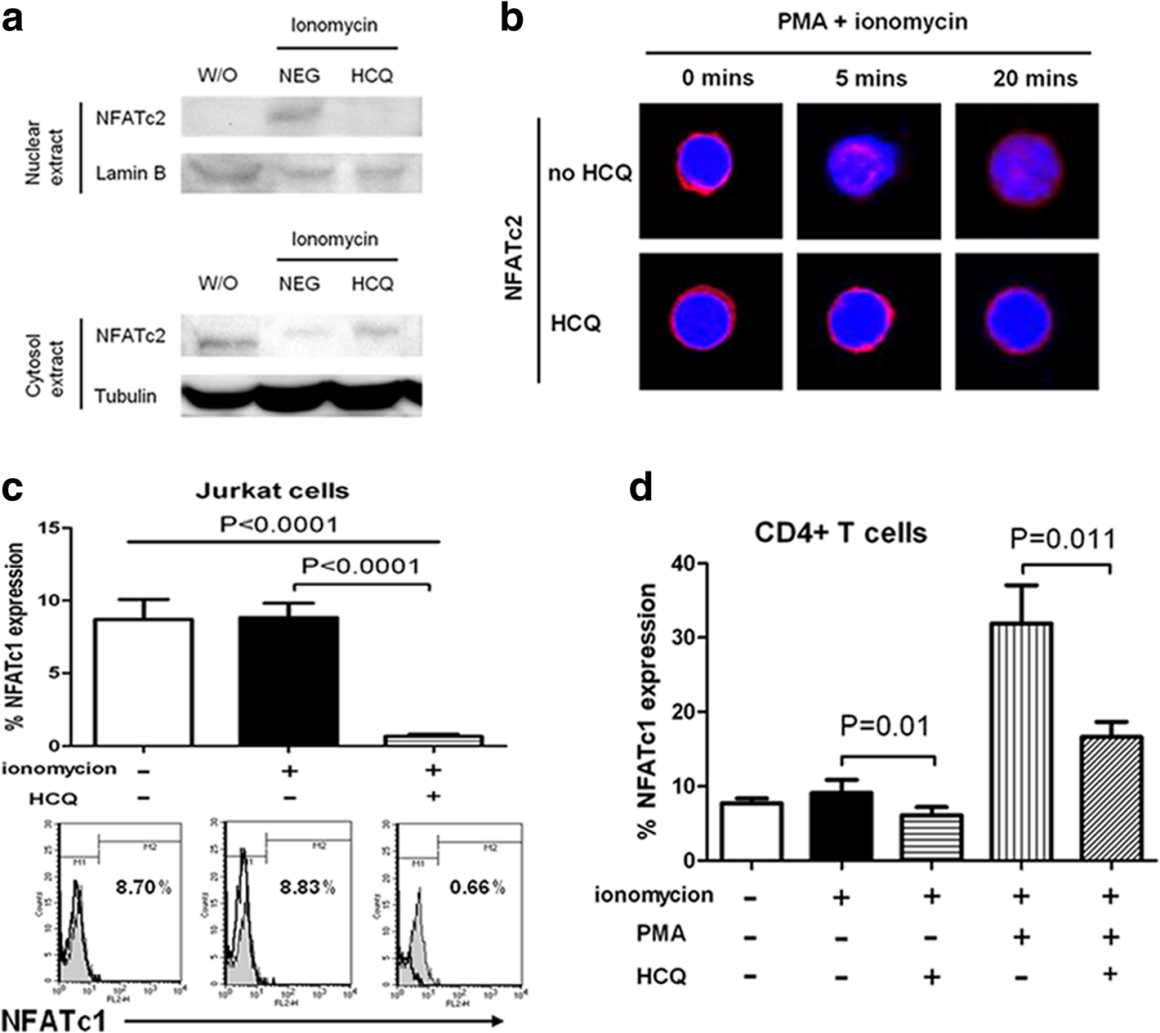
HCQ treatment decreases nuclear translocation and expression of NFAT. **a** Jurkat cells were pretreated without (*W/O*) or with 10× concentration of HCQ (1 × = 600 ng/ml) for 24 hours and then stimulated with ionomycin (1 μg/ml) for 2 hours or left unstimulated. Nuclear and cytoplasmic fraction protein of cells was extracted. Western blot analysis was performed using nuclear protein and antibodies specific for NFATc2 and lamin (*upper panel*). Western blot analysis using cytoplasmic protein and antibodies specific for NFATc2 and tubulin was also performed (*lower panel*). All data shown are representative of three independent experiments with similar results. **b** CD4^+^ T cells were left unstimulated or stimulated with ionomycin (1 μg/ml) and PMA (50 ng/ml) for 5 or 20 min in the presence or absence of the preceding 10× concentration HCQ, as indicated. NFATc2 localization was analyzed using immunocytochemistry and confocal microscopy (original magnification × 1000). All images shown are representative of four independent experiments with similar results. **c** Jurkat cells were pretreated with or without 10× concentration HCQ for 24 hours and were then activated by ionomycin (5 μg/ml) for 2 hours. NFATc1 expression was measured by flow cytometry. (*n* = 11). **d** Purified CD4^+^ T cells from SLE patients were treated without or with 10× concentration HCQ for 24 hours and activated without or with ionomycin (5 μg/ml) or ionomycin (1 μg/ml)/PMA (50 ng/ml) for 2 hours. Flow cytometry was used to measure NFATc1 expression (*n* = 8). *HCQ* hydroxychloroquine, *NFAT* nuclear factor of activated T cells, *PMA* phorbol 12-myrisate 13-acetate, *NEG* no HCQ treatment

NFAT is calcineurin dependent and a secondary messenger downstream of the Ca^2+^ signaling pathway. Previous studies found that increased and prolonged CD154 expression in SLE patients was associated with enhanced NFATc2-mediated CD154 gene transcription [14]. When compared with control, more nuclear translocation of NFATc2 was found in SLE patients and was associated with higher CD154 expression [19]. In the data already presented, we confirmed that HCQ blocked ionomycin induction of CD154 expression and intracellular Ca^2+^ response. Therefore we wanted to validate whether HCQ could inhibit nuclear translocation of NFATc2. Because a large amount of cells was needed for western blot analysis of NFAT, the immortalized cell line of human T lymphocyte cells (Jurkat cells) was used [43]. After pretreatment with vehicle or 10× standard concentration of HCQ (=6000 ng/ml) for 24 hours and stimulation with ionomycin for 2 hours, the nuclear and cytoplasmic fractions of Jurkat cells were extracted. Western blot analysis was used for examination of the nuclear and cytoplasmic levels of NFATc2. The results showed that the poststimulated NFATc2 level decreased in the nucleus fraction and increased in the cytoplasmic fraction upon HCQ pretreatment when compared with vehicle (Fig. 5a). We demonstrated that HCQ had an inhibitory effect on poststimulated nuclear translocation of NFATc2 in Jurkat cells. Therefore HCQ could abrogate NFATc2 nuclear translocation. To further confirm the inhibitory effect of HCQ on NFATc2 nuclear translocation, we performed immunocytochemistry analysis in purified lupus CD4^+^ T cells. In the absence of ionomycin stimulation and HCQ treatment, NFATc2 (red) remained in the cytoplasm (Fig. 5b). However, stimulation with ionomycin and PMA led to an increase of NFATc2 translocation into the nucleus. Then we analyzed nuclear translocation of NFATc2 after pretreatment with HCQ at 10× standard concentration (=6000 ng/ml). Upon stimulation, lesser NFATc2 nucleus translocation was found under HCQ pretreatment when compared with negative control (Fig. 5b). These results indicate that HCQ suppresses CD154 expression by inhibiting nuclear translocation of NFATc2 in lupus CD4^+^ T cells.

Previous studies revealed that NFATc2 is abundant in the cytoplasm of resting T cells, whereas NFATc1 expression is induced by nucleus translocated NFATc2 during T-cell activation [44, 45]. NFATc1 is more predominant than NFATc2 after T-cell activation in pediatric SLE patients and is also responsive for CD154 expression [3, 11, 14]. In the data already presented, we confirmed that HCQ treatment significantly decreased NFATc2 nuclear translocation. Therefore, we examined whether HCQ could inhibit NFATc1 expression. We first applied the experiments on Jurkat cells. Cells were first treated with HCQ at 10× standard concentration (=6000 ng/ml) for 24 hours and then stimulated with ionomycin for 2 hours. The results showed that HCQ significantly decreased NFATc1 expression when HCQ preceded ionomycin treatment (Fig. 5c). Next, using purified CD4^+^ T cells from lupus patients, we also found that HCQ pretreatment significantly decreased NFATc1 expression in activated CD4^+^ T cells (Fig. 5d). Because the percentage of NFATc1-expressing T cells was lower after ionomycin stimulation, we performed the same experiments using a stronger stimulus with ionomycin and PMA. A similar result was found that the elevated expression of NFATc1 after stimulation was inhibited by HCQ (Fig. 5d). Therefore, combined with our previous results, we were convinced that HCQ attenuated CD154 expression in SLE patients partially by inhibiting NFAT signaling.

### HCQ treatment did not inhibit CD154 expressed through IL-15-mediated STAT5 signaling pathway

**Fig. 6:**
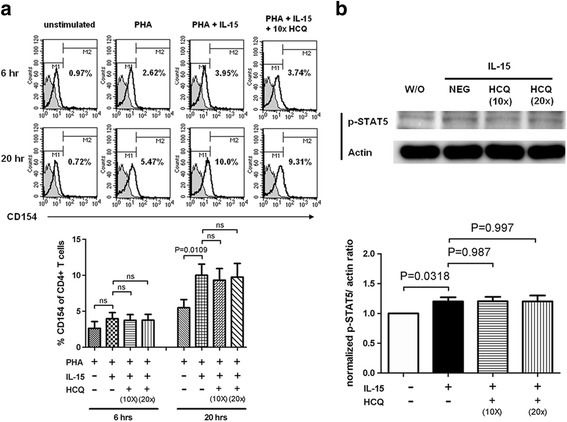
HCQ treatment did not inhibit CD154 expressed through the IL-15-mediated STAT5 signaling pathway. **a** CD4^+^ T cells from SLE patients were cultured in different concentrations of HCQ (1 × = 600 ng/ml) for 24 hours and then activated with PHA (1.5 μg/ml), IL-15 (75 ng/ml), or a combination for 6 or 20 hours. Flow cytometry was used to measure CD154 expression. Histogram plots shown are representative of five similar experiments (*upper panel*). Flow cytometric results are shown as a percentage of CD154 expression of CD4^+^ T cells. Data are mean ± SEM of duplicate samples and are representative of five independent experiments (*lower panel*). **b** Jurkat cells were pretreated with the indicated concentration of HCQ (1 × = 600 ng/ml) for 24 hours and then stimulated with IL-15 (75 ng/ml) for 20 min or left unstimulated (*W/O*). Western blot analysis was performed using cell extracts and antibodies specific for phosphorylated STAT5. Results shown are representative of six similar experiments (*upper panel*). Western blot results are shown as a normalized phosphorylated STAT5/actin ratio. Data are mean ± SEM of duplicate samples and are representative of six independent experiments (*lower panel*). *HCQ* hydroxychloroquine, *IL* interleukin, *ns* not significant, *PHA* polyhydroxyalkanoates, *STAT5* signal transducer and activator of transcription 5

Overexpression of IL-15 was noted in SLE patients [22, 23]. IL-15 stimulation can induce prolonged expression of membranous CD154 in T cells through the STAT5 signaling pathway [21]. We investigated whether the HCQ treatment could abrogate IL-15-mediated CD154 expression. Purified CD4^+^ T cells were first treated with vehicle or HCQ of various concentrations (1 × = 600 ng/ml) for 24 hours and then stimulated with PHA, recombinant IL-15, or a combination for 6 or 20 hours. CD154 expression of T cells was analyzed by flow cytometry. The results showed that the prolonged CD154 expression was not attenuated by HCQ pretreatment (Fig. 6a). Furthermore, we wanted to examine whether HCQ could inhibit STAT5 signaling. Jurkat cells were treated without or with various concentrations of HCQ for 24 hours and stimulated with recombinant IL-15 (75 ng/ml) for 20 min. Western blot analysis was used for measuring the level of phosphorylated STAT5 in cell extracts. We found that the increased phosphorylated STAT5 after IL-15 stimulation did not change upon HCQ pretreatment (Fig. 6b). Therefore the results revealed that HCQ treatment did not inhibit membranous CD154 expressed through the IL-15-mediated STAT5 signaling pathway.

## Discussion

**Fig. 7:**
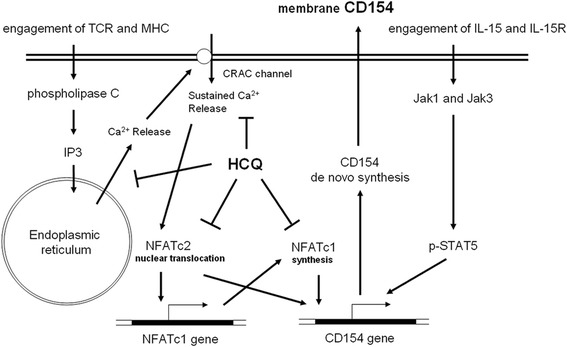
Proposed model for HCQ downregulation of CD154 expression. In this model based on our results, HCQ inhibits membrane CD154 expression through blocking intracellular Ca^2+^ signaling, thus keeping the NFAT transcription factor in an inactive state. *TCR* T-cell receptor, *MHC* major histocompatibility complex, *IP3* inositol triphosphate, *CRAC* Ca^2+^ release-activated Ca^2+^, *NFAT* nuclear factor of activated T cells, *HCQ* hydroxychloroquine, *IL* interleukin, *STAT5* signal transducer and activator of transcription 5

In this study, we found that HCQ could attenuate membranous CD154 expression in activated CD4^+^ T cells from SLE patients via inhibition of the NFAT signaling pathway (Fig. 7). In SLE patients, CD154 was overexpressed and correlated with disease severity [7, 11, 14, 17, 26]. In our study, we also found that the poststimulated CD154 expression was higher in patients with higher SLE disease activity (Fig. 1). To date, although various previous clinical studies have shown that HCQ is beneficial in reducing the severity and flare of SLE disease, its mechanisms remain largely unknown [46, 47]. We speculated that the HCQ effect in treating SLE patients might be partially due to diminished CD154 expression and blocking the NFAT pathway.

CD154 expression is dependent on the calcium–NFAT pathway. Previous studies showed that the calcium and NFAT signaling response after activation was higher in lupus patients than in healthy controls [14, 20]. In addition, the IL-15–STAT5 signaling pathway is also associated with CD154 expression [21]. Higher serum IL-15 and higher IL-15 secretion from poststimulated T cells were found in SLE compared with the control [22, 23]. Thus, previous studies have shown that poststimulated CD154 expression in T cells of SLE patients is higher than that of healthy controls [14, 15]. T cells from healthy donors have a low level of CD154 expression after stimulation [15]. In our study, we also found that higher CD154 was expressed in lupus patients than in healthy controls despite stimulation with various durations or concentrations (Figs. 1 and 2b).

Previous studies showed a clinical association between the poststimulated CD154 expression and ESR and lupus nephritis, but not medications or level of autoantibodies in SLE patients [14, 28]. Our study also revealed association with lupus nephritis, but not with ESR, medications, and autoantibodies (Fig. 1). The clinical ESR level fluctuated and was easily affected by clinical conditions (such as fever, dehydration, infection) and medication (such as steroid). Furthermore, we found that the CD154 level was higher in patients with lupus nephritis than without nephritis, although not significantly (Fig. 1). The definition of nephritis is different between our study and the previous study. Mehta et al. used a history of biopsy-proven glomerulonephritis as the definition of nephritis [14, 26].We defined proteinuria when blood sampling (daily urine protein > 500 mg/day) indicated nephritis. Besides, some factors are also related to the differences, including no available data of biopsy,pathological type, severity of nephritis and concurrent drugs. Finally, distinct stimulation duration and agents in our study may cause the different results [14, 28]. We use ionomycin stimulation alone for 6 hours but not ionomycin plus PMA stimulation for 24 hours in the previous study [14]. Downregulation of expressed CD154 was noted 24 hours after ionomycin stimulation (Fig. 2b, left panel). The intensity of CD154 expression was lower in our study.

Stimulation with ionomycin and PMA can induce higher CD154 expression than with ionomycin alone. However, ionomycin and PMA regulate the expression of CD154 in different mechanisms [41]. Ionomycin regulates membranous CD154 expression through the calcium pathway and PMA regulates soluble CD154 by the PKC-dependent pathway. PMA induced shedding of membranous CD154 from the T-cell surface, resulting in soluble CD154. In this study, we wanted to investigate the effect of HCQ on the expression of membranous CD154 and focused on the calcium pathway. Therefore we used ionomycin alone without PMA to stimulate cells. Besides, we also measured soluble CD154 after ionomycin stimulation in our experiments (data not shown) and found that soluble CD154 was not detectable after ionomycin stimulation, compatible with previous studies [41]. In contrast, if stimulated with ionomycin + PMA, soluble CD154 was induced and could be inhibited by HCQ pretreatment (data not shown).

In the previous study, the prolonged expression of CD154 after stimulation was noted in T cells isolated from SLE patients taking HCQ [14]. In our study, HCQ inhibited CD154 expression in purified T cells from SLE patients. The different results were due to different experiment methods. In-vitro experiments of HCQ were performed in our study, and ex-vivo experiments were done in previous studies [14]. We used HCQ pretreatment before T-cell stimulation. HCQ in the culture medium had a sustained effect on T cells. In the previous study, they used ex-vivo T cells from SLE patients taking HCQ. When they stimulated T cells, there was no HCQ in the culture medium.

HCQ has been reported to induce apoptosis of peripheral blood T cells from SLE patients [48]. Obvious cell apoptosis was found when treated with HCQ of 30 μg/ml for 24 hours in the previous study [48]. However, cell viability of more than 90% was noted. In our study with purified CD4^+^ T cells, CD154 expression was significantly inhibited when pretreated with HCQ of 5× standard concentration for 24 hours (Fig. 3a). The expressed CD154 reduced by 20% when compared with vehicle. However, the bioactivity of cells only reduced by less than 10% when 5× HCQ was used (Fig. 3b). Therefore the toxicity of HCQ was limited when a lower concentration was used. In addition, in the IL-15 study, CD154 expression did not decrease even when HCQ of 20× standard concentration was used for pretreatment (Fig. 6a). Besides, we used gated CD4^+^ cells to analyze the CD154 expression by flow cytometry. The previous study showed that apoptotic cells lost the CD4 surface marker while viable T cells keep cell surface CD4 expression [48]. Therefore, analysis of gated CD4^+^ cells in our study decreased the interference of apoptotic cells.

Our data demonstrated that HCQ can attenuate NFATc2 nuclear translocation and NFATc1 expression and can inhibit sustained Ca^2+^ storage release from the endoplasmic reticulum (Figs. 4 and 5). The Ca^2+^ signaling pathway was important for T-cell activation. Previous studies also showed that HCQ could block activation of T cells from healthy controls and downregulate the activation marker CD69 in vitro [36]. This HCQ effect was due to inhibition of calcium mobilization in a concentration-dependent manner [36]. However, in SLE patients, CD154 overexpression was not simply due to the global activation states of T cells because the activation markers, CD69 and CD25, were not upregulated in SLE [15, 17]. Therefore, as in the previous report, overexpression of CD154 in SLE was largely due to upregulation at the transcriptional level [14]. NFAT is the most important transcription factor for CD154 synthesis [3, 49]. In a previous study, CD154 levels increased in CD4^+^ T cells from pediatric lupus patients [14]. The enhanced CD154 expression was due to upregulated transcription rates and increased NFAT activity. We reported that inhibition of CD154 expression by HCQ in T cells from SLE patients may be due to directly attenuating the CD154 transcription by inhibiting NFATc2 translocation and NFATc1 expression, not simply due to blocking T-cell activation (Fig. 5). These results may partially explain its efficacy in many autoimmune diseases.

Increased IL-15 production was noted in serum and activated T cells of SLE patients [22, 23]. The IL-15-mediated STAT5 signaling pathway was found to participate in the prolonged CD154 expression [21]. We demonstrated that the HCQ treatment did not attenuate the CD154 expression and STAT5 signaling after IL-15 stimulation (Fig. 6). This finding was not compatible with a previous report that the STAT5 expression level in T cells was lower in SLE patients receiving HCQ treatment [50]. However, in that previous study, STAT5 levels were associated with SLE disease activity. The lower STAT5 in the HCQ treatment group may be due to decreased disease activity but not directly inhibited by HCQ.

The etiology of SLE is mainly due to production of pathogenic autoantibodies. This process requires T-cell help, along with the interaction of CD154 (on the T-cell surface) with CD40 (on the B-cell surface), which is critical for B-cell survival and proliferation, memory generation, and, most importantly, antibody synthesis [1, 3, 11]. Furthermore, recent data from SLE patients and murine lupus models have demonstrated that increased and prolonged expression of membranous CD154 enhanced its capacity to mediate excessive B-cell activation and autoantibody formation [7, 14, 26]. Therefore, at present, there is extensive research into CD154 as a target for therapy in SLE. Both cyclosporine and dipyridamole could inhibit CD154 expression and were demonstrated effective in SLE treatment [10]. Treatment with anti-CD154 mAbs prior to disease onset in SLE mouse models prolongs survival, prevents development of proteinuria, ameliorates kidney disease, and decreases anti-DNA autoantibody titers [16, 27, 51, 52]. Moreover, in the diagnosed SLE, anti-CD154 treatment slows down disease progression, reverses proteinuria, and induces remissions in mice even when lupus nephritis has already developed. HCQ was used to treat SLE for decades and has proven to have a significant effect in disease control and the maintenance of remission [46]. Our study demonstrates that HCQ could abrogate CD154 expression through inhibiting the NFAT pathway in T cells; to our knowledge, this is a newly reported SLE therapeutic effect of HCQ, which may partially explain why HCQ has been well suited for SLE drug therapy.

## Conclusions

HCQ treatment has a dose-dependent suppressive effect on CD154 expression in T cells of SLE patients because of its inhibitory effect on NFAT, rather than the STAT5 signaling pathway. These results indicate why HCQ is a first-line therapy as an anti-rheumatic drug, and further provides mechanistic evidence for HCQ being highly recommended for the treatment of SLE.

## Abbreviations

ESR: Erythrocyte sedimentation rate
HCQ: Hydroxychloroquine
IL: Interleukin
NFAT: Nuclear factor of activated T cells
SLE: Systemic lupus erythematosus
SLEDAI: SLE Disease Activity Index
STAT5: Signal transducer and activator of transcription 5
